# Actinomycetes as host cells for production of recombinant proteins

**DOI:** 10.1186/1475-2859-4-7

**Published:** 2005-03-23

**Authors:** Nobutaka Nakashima, Yasuo Mitani, Tomohiro Tamura

**Affiliations:** 1 Proteolysis and Protein Turnover Research Group, Research Institute of Genome-based Biofactory, National Institute of Advanced Industrial Science and Technology (AIST), 2-17-2-1 Tsukisamu-Higashi, Toyohira-ku, Sapporo 062-8517, Japan; 2 Center for Genomics and Bioinformatics (CGB), Karolinska Institute, Berzelius väg 35, Stockholm 171 77, Sweden; 3 Laboratory of Molecular Environmental Microbiology, Graduate School of Agriculture, Hokkaido University, Kita-9, Nishi-9, Kita-ku, Sapporo 060-8589, Japan

## Abstract

Actinomycetes (*Actinobacteria*) are highly attractive as cell factories or bioreactors for applications in industrial, agricultural, environmental, and pharmaceutical fields. Genome sequencing of several species of actinomycetes has paved the way for biochemical and structural analysis of important proteins and the production of such proteins as recombinants on a commercial scale. In this regard, there is a need for improved expression vectors that will be applicable to actinomycetes. Recent advancements in gene expression systems, knowledge regarding the intracellular environment, and identification and characterization of plasmids has made it possible to develop practicable recombinant expression systems in actinomycetes as described in this review.

## Review

As a result of the sequencing of entire genomes of more than 100 organisms, the number of open reading frames with unknown functions has increased. To characterize such proteins biochemically and structurally, it is important to obtain substantial amounts of recombinant proteins. *Escherichia coli*, a Gram-negative γ-proteobacterium, is the most commonly used host bacterium for the large-scale production of recombinant proteins. However, the expression and isolation of all the proteins in *E. coli *is difficult on account of the problems of insolubility, cytotoxicity, post-translational modifications, or inefficient translation.

In order to overcome these problems, host-vector systems other than *E. coli *have also been developed in both prokaryotic and eukaryotic cells. *Bacillus *spp. (e.g., *B. brevis *and *B. subtilis*) and *Lactococcus lactis *are Gram-positive bacteria with low G + C content and are often used to secrete expressed proteins into the culture media [1-5]. Secretion prevents the local accumulation of the recombinant proteins and this occasionally aids in correct protein folding [6]. *Bacillus brevis *has an extremely high capacity of protein secretion and is being used for the expression of prokaryotic and eukaryotic proteins on an industrial scale [2]. *Lactococcus lactis *can also be used for the secretion of recombinant proteins and these proteins can be used directly in food applications [5]. Eukaryotic cells such as yeast cells, insect cells, or immortalized cell lines are particularly useful for the expression of proteins that undergo post-translational modifications [7].

In actinomycetes (Gram-positive bacteria with a high G + C content), the genera *Streptomyces*, *Rhodococcus*, *Corynebacterium*, and *Mycobacterim *have received an increasing amount of attention, particularly in the industrial fields. They exhibit potential advantages in the synthesis of secondary metabolites of industrial and medical importance, in the production of amino acids by fermentation, and in bioconversion processes. There have also been several host-vector systems developed for actinomycetes [8,9], although further improvements were needed to provide highly inducible and tightly regulated promoters, broad-host range vectors, and high producibility of recombinant proteins. Recently, improvements in the host-vector systems in this class of bacteria were reported, thereby making it possible to obtain significant amounts of recombinant proteins under strictly regulated promoters [10-12]. Here, we review the host-vector systems, particularly expression vectors, in actinomycetes and also describe the benefits and future possibilities of the system.

### Why actinomycetes?

We can highlight two striking characteristics of actinomycetes as host cells. First, they exhibit a unique metabolic diversity and enzymatic capabilities. The compounds they produce as secondary metabolites are valuable for industrial and pharmaceutical purposes [13], and the enzymes themselves are also valuable. For example, *Streptomyces *spp. produce various types of antibiotics [14] and some *Rhodococcus *spp. are being used for the industrial production of acrylamide [15]. Historically, the host-vector systems in actinomycetes have been developed to obtain such enzymes in large quantities and/or to manipulate the metabolic pathway involved in the production of antibiotics [8,9]. Second, actinomycetes are expected to have different intracellular milieu as compared to conventional host cells such as *E. coli*. Until recently, no host cell from which all the proteins can be universally expressed in large quantities has been found. Therefore, it is important to provide a variety of host-vector systems (expression systems) in order to increase the opportunities to screen for the most suitable expression conditions or host cell.

It is important to select an appropriate promoter for high-level protein expression, and generally, an inducible promoter is more preferable than a constitutive promoter [8,10]. Several reports used well characterized promoters of the nitrilase gene [10], acetoamidase gene [16], and *tipA *[11,12,17]. In *S. coelicolor*, the expression vector containing *act*I/*act*III promoter was induced during the transition from the growth to the stationary phase to successfully produce polyketido synthases [18]. A derivative of this vector can be used with other actinomycetes [19], thereby expanding the application of the expression system. In *M. smegmatis*, novel strong constitutive promoters were identified using a genomic library fused to promoterless green fluorescent protein in combination with a fluorescence-associated cell sorting technique [20]. On the other hand, the mutagenesis of the repressor gene is another possible strategy in which the constitutively expressed temperature-sensitive repressor protein is unable to repress the target gene expression at a high temperature [21,22]. In this review, we have focused on the systems using heterologous promoters to drive strong expression and some examples of such vectors of actinomycetes are summarized in Table 1. In the next sections, several recent topics are discussed in detail.

### Streptomyces species

Among the *Streptomyces *spp., *S. lividans *has been extensively utilized as a potential host for both cytoplasmic and secreted protein production because it lacks restriction systems that are generally present in other *Streptomyces *and prevent the genetic manipulation of host cells [23]. In addition, *S. lividans *also exhibits a very low endogenous extracellular proteolytic activity [23].

Recently, Herai et al. reported an expression vector that functions in several *Streptomyces *spp. [10]. The vector carries the nitrilase gene promoter (P*nitA*) originating from the nocardioform actinomycete *Rhodococcus rhodochrous *J1 [15]. The expression is tightly regulated and strongly induced only in the presence of the inducer ε-caprolactam. These researchers expressed several bacterial genes and estimated that up to ~40% of all soluble protein comprised a target protein and that up to 396 mg of the protein per liter of culture media was produced (e.g., the *Streptomyces *inducible expression system had thus far expressed a maximum of 38 mg of the protein per liter of the culture media [10,24]). However, the report does not refer to secreted proteins or the production of proteins originating from sources other than bacteria. This hyperinducible expression vector can be further improved to enable protein secretion and can be used to express higher eukaryotic proteins. This vector may also enable rapid progress in genome mining and the production of natural-product gene clusters such as those identified for enediyne antibiotics [25].

An increasing number of studies over the past years have reported *Streptomyces *as an ideal host for the production of secreted proteins. Signal peptides are an important factor for improving the efficiency of secreted protein production, and are extensively studied via mutagenic approaches [26]. Several *Streptomyces *secretion systems have successfully produced eukaryotic proteins [27-29]. The soluble form of human CD4 was efficiently produced using *S. lividans *as a host cell. Over 300 mg of protein was produced per liter culture by using pLTI-CD4 containing *S. longisporus *serine protease inhibitor gene promoter and secretion signals [27].

### Rhodococcus species

In *Rhodococcus*, several cloning vectors have been developed since the first report on *E. coli*-*Rhodococcus *shuttle vector [30], and recently, versatile expression vectors have been constructed [11,12]. The expression levels of proteins from these vectors are not as high as that from the expression system in *E. coli *(a maximum of 10 mg of protein per liter of culture media). However, in *Rhodococcus*, proteins can be expressed over a wide range of temperatures – from 4°C to 35°C [11,12] – because some *Rhodococcus *cells are psychrotrophic. When the thiostrepton inducible *tipA *promoter was used, several milligrams of mouse protein per liter of culture media could be expressed even at 4°C [11].

Most of the recombinant protein expression systems established until recently can only be used within the range of 10°C to 37°C. For example, *E. coli *is a mesophilic bacterium that grows at temperatures ranging from 18°C to 37°C, and recombinant protein expression has been carried out in a similar range of growth temperatures [31]. It is commonly known that a lower temperature is often more preferable for the production of recombinant proteins [32]. Some mouse proteins that could not be expressed in *E. coli *could be expressed in *Rhodococcus *at 4°C [11]. The expression at lower temperatures is expected to be effective in producing proteins that damage the host cell, because enzymatic activities of such proteins can be suppressed.

In the case of *R. erythropolis*, the mycolic acid composition of the host cells makes it difficult to disrupt the cell walls and necessitates an approach for the modification of the host cells to simplify recombinant-protein extraction procedures [33]. The authors reported lysozyme-sensitive mutants that can be lysed by the addition of 12.5 μg ml^-1 ^lysozyme, while the wild type is resistant to over 1 mg ml^-1 ^lysozyme. *Rhodococcus *spp. are tolerant to various organic solvents and toxic chemicals. A highly efficient bioconversion process can be achieved by the combination of the expression system and this characteristic feature of *Rhodococcus *spp.

### Corynebacterium and Mycobacterium species

*Corynebacterium *and *Mycobacterium *spp. are phylogenetically closely related to *Rhodococcus *spp. [34]. *C. glutamicum *is used for the industrial production of L-glutamate, while *C. diphtheriae *is the causative agent of diphtheria [35]. Among the *Mycobacterium *spp., the fast-growing non-pathogenic *M. smegmatis *is widely used as a model species [36]. Microorganisms such as *M. tuberculosis *and *M. leprae*, which are highly virulent human pathogens, are also well characterized species [8]. Spratt et al. identified strong expression promoters and demonstrated that one of them enabled the production of approximately 125 μg protein per milligram cell lysate [20]. The techniques developed by the authors for identifying the above mentioned novel promoters may be useful, although the technique is only applicable to constitutive promoters. As shown in Table 1, some other expression vectors in these species are used to express homologous and/or heterologous proteins.

## Conclusion

When expressing recombinant proteins, it is often recommended to use host cells that are phylogeneticaly closely (ideally, identical) related to the origin of the protein of interest. This is due to the similarity in frequency of codon usage, compatibility with machineries of translation and molecular chaperones, and/or redox states of the cells. Hence, when expressing higher eukaryotic proteins, in principle, using higher eukaryotes as hosts is ideal but it often results in low yields, and furthermore, is expensive and time consuming.

The host-vector systems of actinomycetes are suitable for expressing proteins of actinomycetes and proteins from closely related organisms as well as from higher eukaryotes. However, further development of host-vector system in actinomycetes is required, particularly with respect to the modification of host cells. This includes improvement in stability and easy maintenance of foreign genes (e.g., integration of plasmids), removing host proteins that hamper production (e.g., knock-out of proteases) either at the gene level or during the extraction of proteins.

## Figures and Tables

**Table 1:** Expression vectors in actinomycetes

Vectors	Inducers	Location	Comments	Ref.
***Streptomyces***				
pSH19	ε-caprolactam	cytoplasm	high-level expression	[10]
pULHis2	thiostrepton	cytoplasm		[17]
pRM5	-	cytoplasm	growth phase-dependent expression	[18]
pITS-vectors	-(constitutive)	cytoplasm		[21]
pLTI-CD4	-(constitutive)	secretion	>300 mg l^-1 ^production of CD4	[27]
pSJ205ΔEcA	-(constitutive)	secretion		[28]
pLMS-vectors	-(constitutive)	secretion		[29]
				
***Rhodococcus***				
pTip-vectors	thiostrepton	cytoplasm	expression at low temperatures	[11,12]
pNit-vectors	-(constitutive)	cytoplasm	constitutive type of pTip-vectors	[12]
pNT	-(constitutive)	cytoplasm		[37]
				
***Mycobacterium***				
pJAM2	acetoamide	cytoplasm		[16]
pJEX77	-(constitutive)	cytoplasm		[20]
pJEM15-*traR*^ts107^-P*tra*	high temperature	cytoplasm	expression level is not high	[22]
				
***Corynebacterium***				
pECTAC-K99	-(constitutive)	cytoplasm		[36]
pCGL-vectors	-(constitutive)	secretion		[38]
pWLQ-vectors	IPTG	cytoplasm or secretion		[39]
